# Chromosomal Rearrangements Formed by *rrn* Recombination Do Not Improve Replichore Balance in Host-Specific *Salmonella enterica* Serovars

**DOI:** 10.1371/journal.pone.0013503

**Published:** 2010-10-19

**Authors:** T. David Matthews, Robert Edwards, Stanley Maloy

**Affiliations:** 1 Center for Microbial Sciences, Department of Biology, San Diego State University, San Diego, California, United States of America; 2 Department of Computer Science, San Diego State University, San Diego, California, United States of America; 3 Mathematics and Computer Science Division, Argonne National Laboratory, Argonne, Illinois, United States of America; University of Hyderabad, India

## Abstract

**Background:**

Most of the ∼2,600 serovars of *Salmonella enterica* have a broad host range as well as a conserved gene order. In contrast, some *Salmonella* serovars are host-specific and frequently exhibit large chromosomal rearrangements from recombination between *rrn* operons. One hypothesis explaining these rearrangements suggests that replichore imbalance introduced from horizontal transfer of pathogenicity islands and prophages drives chromosomal rearrangements in an attempt to improve balance.

**Methodology/Principal Findings:**

This hypothesis was directly tested by comparing the naturally-occurring chromosomal arrangement types to the theoretically possible arrangement types, and estimating their replichore balance using a calculator. In addition to previously characterized strains belonging to host-specific serovars, the arrangement types of 22 serovar Gallinarum strains was also determined. Only 48 out of 1,440 possible arrangement types were identified in 212 host-specific strains. While the replichores of most naturally-occurring arrangement types were well-balanced, most theoretical arrangement types had imbalanced replichores. Furthermore, the most common types of rearrangements did not change replichore balance.

**Conclusions/Significance:**

The results did not support the hypothesis that replichore imbalance causes these rearrangements, and suggest that the rearrangements could be explained by aspects of a host-specific lifestyle.

## Introduction

Numerous examples of large-scale chromosomal rearrangements between different strains of the same species or closely related species have been identified [1], [2], [3], [4], [5], [6], [7], [8], [9], [10], [11], [12], [13], [14], [15], [16]. These rearrangements change the order of genes around the chromosome by translocating and inverting chromosomal regions. In addition, some rearrangements are not tolerated, indicating that there are selective forces that limit genome plasticity. Some features of chromosome organization that affect plasticity include the frequency of multiple homologous sequences on the chromosome, gene location and dosage [17], [18], [19], [20], [21], orientation of polarized sequence motifs such as *ter* sites and KOPS (used to terminate DNA replication and direct DNA shuffling by FtzK repectively) [22], [23], [24], [25], and the organization of chromosomal macrodomains [22], [25].

**Figure 1:**
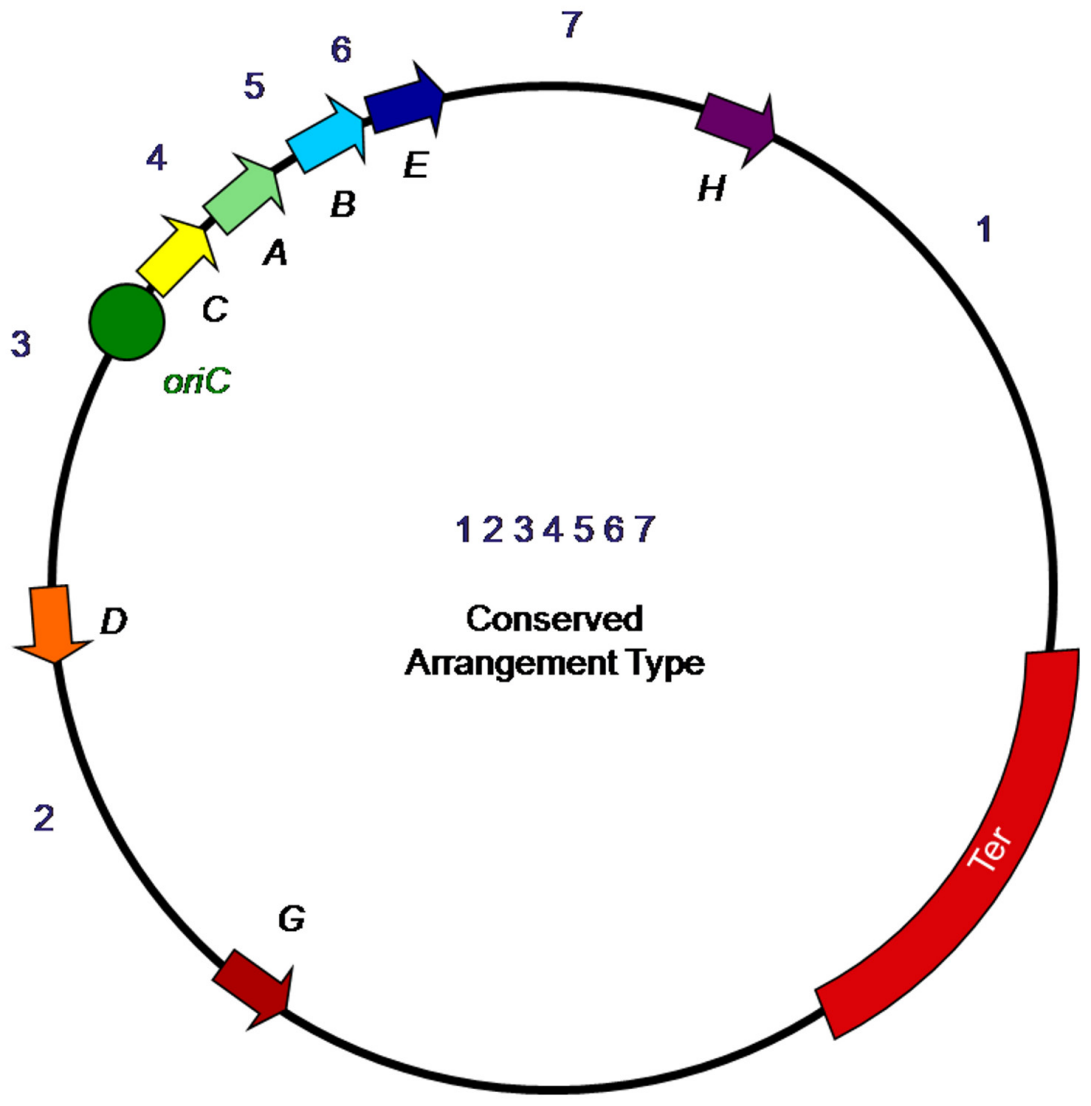
The conserved *rrn* arrangement type 1234567 found in the broad host range serovars of *Salmonella enterica*. The seven *rrn* operons are lettered while the regions in between the operons are numbered. The origin of replication is indicated by a green circle and the terminus region is highlighted in red. Replichores are the chromosomal halves between the origin and terminus of replication.

Another aspect of chromosomal organization that may limit plasticity is replichore balance [26], [27]. In most sequenced bacterial chromosomes, the replichores, opposite sides of the chromosome between the origin of DNA replication and the terminus, are equal in length with the origin 180° around the chromosome from the terminus (Figure 1) [28], [29]. When replichores are of equal length, DNA replication is balanced. Many types of chromosomal rearrangements can make one replichore longer than the other, altering the amount of time required to replicate each replichore. Even though strains having imbalanced replichores are rare because unbalanced replication is thought to affect fitness [22], [26], [30], replichore balance can still vary within the same species [4], [26], [27].

*Salmonella enterica* is an excellent model system for studying both host-specificity and large-scale chromosomal rearrangements in bacterial pathogens. While most of the ∼2,600 *Salmonella* serovars have a broad host range and are capable of infecting a wide variety of animal species, a small number of serovars are host-specific and can only cause disease in one species or in closely related species [31], [32]. Extensive analyses of the genomes of *Salmonella* strains representing broad host range and host-specific serovars has revealed at least two differences between these serovar types: host-specific serovars have a higher number of pseudogenes and their chromosomes are often rearranged [27], [33], [34], [35], [36]. Since the endpoints of these rearrangements mapped to *rrn* operons, they were proposed to occur via homologous recombination between the seven *rrn* operons [37], [38], [39]. The rearrangements are caused by inversions and levitations/translocations of the chromosomal regions between the operons, changing their order from the conserved order found in the broad host range serovars (Figure 1).

Previous work has resolved the genome types of a number of strains belonging to host-specific *Salmonella* serovars [36], [39], [40], [41], [42], [43]. The genome types were defined as the order of restriction fragments (lettered A through G) representing the chromosomal regions between the *rrn* operons, and determined by either physical mapping of partial I-*Ceu*I digest fragments using pulsed field gel electrophoresis, or by analysis of PCR products synthesized using primers specific to the 5′ and 3′ flanking regions of each *rrn* operon. I-*Ceu*I cuts within the 23S rRNA gene and by comparing the size of the partial and complete digest products, 25 naturally-occurring genome types were determined. These genome types were further divided based on the relative orientation of the I-*Ceu*I fragments A (containing the terminus) and C (containing the origin of replication).

It is not clear why the host-specific serovars have rearrangements in contrast to the conserved arrangement type found in the broad host range serovars. One hypothesis proposed by Liu suggests that the insertion of large DNA fragments into the genome, such as pathogenicity islands or prophages, imbalances the replichores, inducing chromosome rearrangements in attempts to restore balance [27], [38], [41]. An alternative hypothesis proposes that aspects of the host-specific serovars' lifestyle, such as the ability to establish chronic long-term infections, either induces the rearrangements or allows them to be tolerated [44].

To test these two hypotheses, the frequency and estimated replichore balance of naturally-occurring arrangement types was compared to the theoretical possibility. Natural arrangement types were identified by compiling data from previous studies, as well as resolving the arrangement type of an additional 22 fowl-specific serovar Gallinarum strains. The naturally-occurring arrangement types were then compared to all possible theoretical arrangement types to answer the question: out of the theoretically possible arrangement types, which ones naturally occur and which ones do not? While naturally-occurring arrangement types can be physically mapped, theoretical arrangement types cannot. This problem was circumvented by developing a replichore balance calculator that estimates the balance of both theoretical and naturally-occurring arrangement types. Naturally-occurring arrangement types were further classified depending on which types of rearrangements took place. The estimated replichore balance of natural and theoretical arrangement types was examined and grouped into four classes based on how much imbalance was estimated. The results support the hypothesis that rearrangements accumulate in host-specific *Salmonella* serovars as a consequence of lifestyle and not from replichore imbalance.

## Results

### *rrn* Arrangement Types

Due to the factorial aspect of the total number of arrangements possible by recombination between *rrn* operons, it is easier to describe the rearrangements in terms of numbers (arrangement types) instead of letters (genome types). For example, the conserved chromosome arrangement type found in the broad host range serovars is described as arrangement type 1234567 instead of genome type 1 with an I-*Ceu*I fragment order of BCDEFG and an A+/C+ orientation. This description of the arrangement type is based on the relative order of the chromosomal regions between the *rrn* operons, starting with Region 1 (containing the terminus) and proceeding clockwise around the chromosome (Figure 1).

To determine the number of possible arrangement types, the limitations of rearrangements due to *rrn* recombination must be considered. As intrareplichore inversions are restricted by the direct repeat nature of the *rrn* operons on each replichore, the number of possible arrangement type combinations is: 6!×2 = 1,440. This number is based on the combinations possible when rearranging the order of the six chromosomal regions surrounding Region 3 (containing the origin of replication) plus the same number with Region 1 inverted (designated 1′). While Region 3 can undergo inversions, under this scheme of describing arrangement types, such an inversion changes the order of the other regions surrounding Region 3 instead of having to describe another set of arrangement types with an inverted Region 3. For example, inversion of Region 3 by recombination between *rrnC* and *rrnD* would change the conserved arrangement type 1234567 to 1′765432.

### Naturally-occurring Arrangement Types

**Figure 2:**
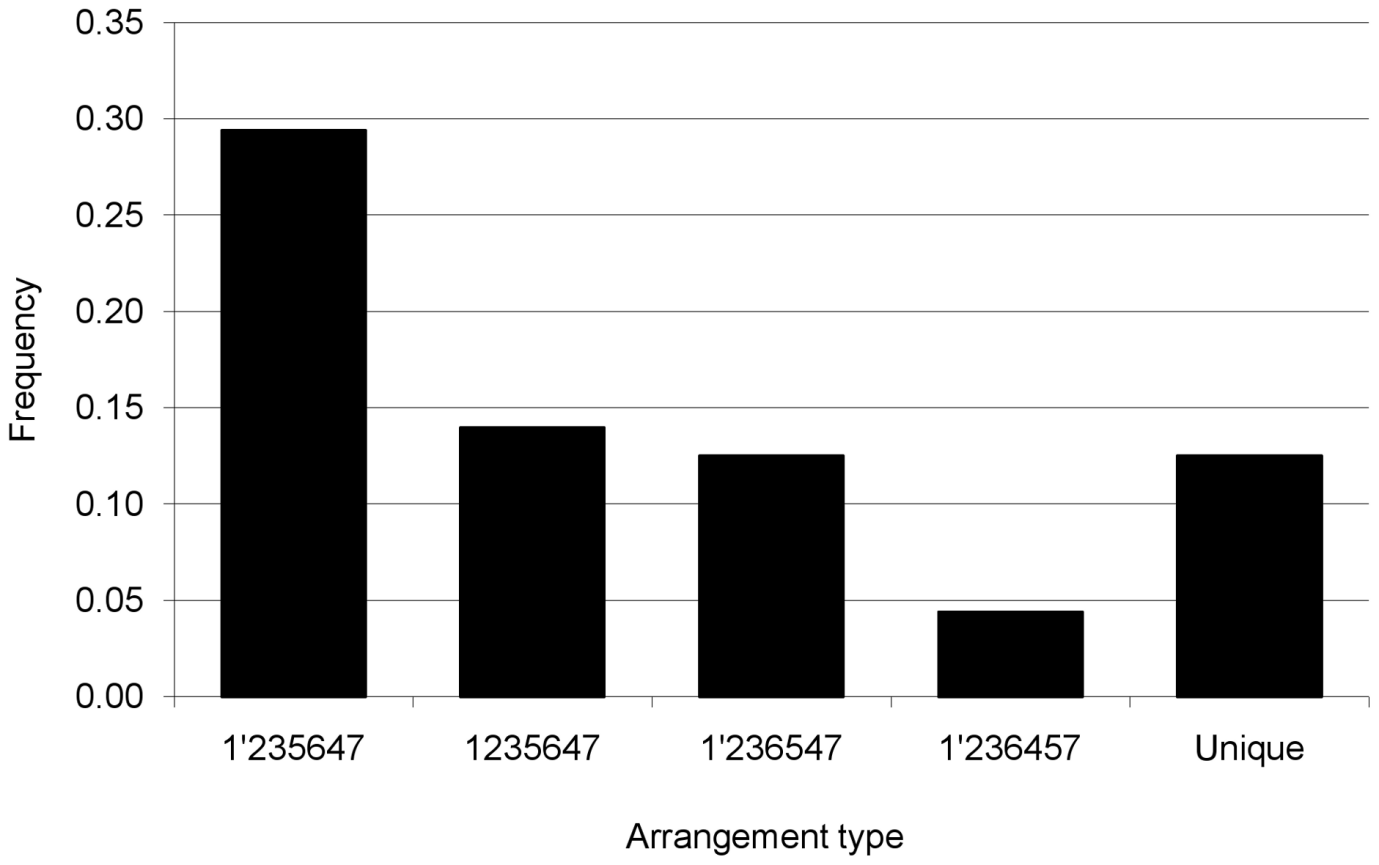
Frequency of most common and unique arrangement types in of *Salmonella enterica* sv. Typhi. Data compiled from 136 Typhi strains previously characterized [44]. 1′ indicates that region 1 is in the inverted orientation. In the two most common arrangement types, Region 4 translocated in between Region 6 and Region 7. The two other common arrangement types had an interreplichore translocation of Region 6 next to Region 3. Except for arrangement types that are unique (only found in one strain), the frequencies of other less common arrangement types are not shown.

Previously the genome types of 136 Typhi strains were determined [40]. After converting the genome types of these strains to arrangement types, 32 arrangement types were found to occur naturally in Typhi. The most common Typhi arrangement type identified was 1′235647, followed by 1235647 (Figure 2). These two arrangement types had a translocation of Region 4 into the *rrnE* operon, and were found in 43% of the strains. The most-common rearrangement found in 74% of the strains analyzed was the inversion of Region 1 by recombination between the *rrnH* and *rrnG* operons. Eight strains were found to have another inversion due to recombination between the *rrnD* and *rrnE* operons. This inversion results in Regions 2 and 7 switching replichores as well as inverting Region 1 (three strains having this inversion as well as Region 1 in the conserved orientation underwent both of the inversions described above). Almost all the other rearrangements involved translocation of Regions 4, 5, and/or 6 to either one of the *rrn* operons bordering these regions, or to *rrnD* on the opposite replichore next to Region 3. Translocations that moved these regions next to Region 1 were only observed in one strain. Seventeen strains (12.5% of the total) have unique arrangement types.

Other human-adapted *Salmonella* serovars also cause enteric fever and have chromosomal rearrangements due to *rrn* recombination. A small number of strains belonging to the Paratyphi A serovar were found to all contain an inversion of Region 1 due to *rrnG*/*rrnH* recombination, but no other types of rearrangements were noted [45]. The most common arrangement type observed in 8 out of 23 strains of Paratyphi C was 1263457, most likely as a result of an interreplichore translocation of Region 6 [42]. Almost all the rest of the Paratyphi C strains had an intrareplichore translocation of Regions 4, 5, or 6. One exception was a strain that had the unusual arrangement type of 1423657, probably due to an interreplichore translocation of Region 4 in between Regions 1 and 2. Paratyphi B was found to have the conserved arrangement type; however only one strain from this serovar has been characterized [46].

Strains belonging to other host-specific *Salmonella* serovars have also been analyzed to determine their chromosomal arrangement types. The two biovars of the fowl-specific Gallinarum serovar, Pullorum and Gallinarum, cause either pullorum disease or fowl typhoid respectively. Pullorum disease usually infects young chicks and poults, causing diarrhea, and is often fatal, whereas fowl typhoid is a chronic systemic infection that occurs in adults [47], [48]. Nineteen strains belonging to the Pullorum biovar have been previously analyzed to determine their arrangement types [39], [41], [49]. Reanalysis of the data showed that 68% of the strains had the arrangement type 1735462, with the remaining strains having unique arrangement types. All of the strains appeared to have an inversion between *rrnD* and *rrnE* resulting in regions 2 and 7 switching replichores. Two Gallinarum biovar strains have been characterized previously. One had an arrangement type of 1′245637 [39], [43], and the other had an arrangement type of 1′734652 [36].

To determine if other arrangement types occur in these fowl-specific *Salmonella* biovars, the arrangement types of eight Gallinarum and fourteen Pullorum strains were determined using PCR (Table 1). Serotyping was confirmed by assaying motility and the ability to agglutinate preabsorbed antiserum specific for O antigen Group D_1_ Factor 9. All 22 strains were non-motile and agglutinated the antiserum, confirming the strains were serovar Gallinarum. The two biovars were distinguished using Moeller decarboxylase broth supplemented with ornithine [50]. All the biovar Pullorum strains were able to decarboxylate ornithine while all the biovar Gallinarum strains did not. Two biovar Gallinarum strains had the conserved arrangement found in the broad-host range serovars while six strains each had a unique arrangement type. In TYT3316, the detection of the hybrid *rrnG/D* operon by PCR suggested that in this strain Region 2 integrated into the chromosome by recombination not involving *rrn* operons. The orientation of Region 1 in TYT3335 could not be ascertained from the PCR results. The results from the PCR analysis of the Pullorum strains showed that half of the strains had the previously observed most common arrangement type of 1735462 and half had unique arrangement types. One strain, TYT3345, also contained a duplication of Region 4. These data show that different biovars of the same serovar can differ in their most common arrangement type.

**Table 1:** Chromosomal arrangement types of *Salmonella enterica* sv. Gallinarum strains.

Gallinarum Biovar		Pullorum Biovar	
Strain	Arrangement Type	Strain	Arrangement Type
TYT3313	1234657	TYT3314	1′234657
TYT3315	1′253467	TYT3326	1735462
TYT3316	1′34657+2	TYT3328	1735462
TYT3325	1267354	TYT3329	1735462
TYT3335	1?753246	TYT3331	1′263547
TYT3339	1263457	TYT3340	1736542
TYT3349	1234567	TYT3341	1735462
TYT3350	1234567	TYT3342	1234657
		TYT3343	1735462
		TYT3345	17354462
		TYT3352	1′756342
		TYT3353	1735462
		TYT3354	1267534
		TYT3355	1735462

The 48 naturally-occurring arrangement types were then organized into 11 rearrangement groups based on the most likely types of rearrangements that occurred during their formation from the conserved arrangement type (Table 2). Intrareplichore translocations of Regions 4–6 were the most common type of rearrangement, followed by the inversion of Region 1 through *rrnG*/*rrnH* recombination. Almost two-thirds of all analyzed strains had either one or both of these types of rearrangements. Interreplichore translocations, which can alter replichore balance, were less frequent and occurred in conjunction with intrareplichore translocations and/or inversions. Two types of inversions occur in naturally-occurring strains; the above mentioned inversion of Region 1 and the inversion of Regions 1, 2, and 7 by recombination through the *rrnD* and *rrnE* operons. A number of strains, mostly from the Gallinarum serovar, have both types of inversions, which returns Region 1 to its original orientation even though the flanking *rrn* operons are hybrids.

**Table 2:** Rearrangement groups of naturally-occurring arrangement types.

Rearrangement Group	Type of Rearrangement	# of Arrangement Types	# of Strains	Range of Estimated Replichore Imbalance
1	Intrareplichore translocation	4	42	0.2°
2	Intra- & interreplichore translocations	10	19	3.4–60.7°
3	G/H inversion & intrareplichore translocation	6	83	11.6°
4	G/H inversion & interreplichore translocation	4	4	22.5–34.1°
5	G/H inversion, intra- & interreplichore translocations	6	8	19.2–43.7°
6	D/E inversion	1	3	13.6°
7	D/E inversion & intrareplichore translocation	3	7	13.6°
8	D/E inversion & interreplichore translocation	3	11	21.2–28.5°
9	D/E inversion, intra- & interreplichore translocations	1	1	16.8°
10	Double inversion & intrareplichore translocation	3	22	7.8–17.1°
11	Double inversion & interreplichore translocation	6	7	5.4–21.5°

**Figure 3:**
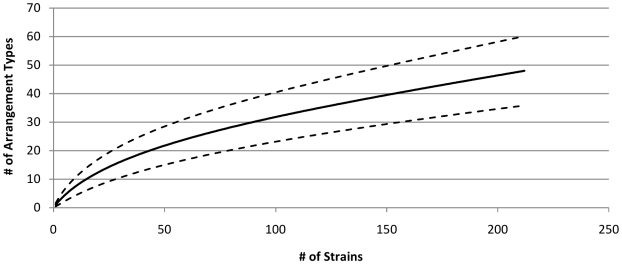
Expected species accumulation curve of naturally-occurring arrangement types. The moment-based estimator of species richness, τ (*h*), was computed using EstimateS version 8.2 [55] with 95% confidence intervals (dashed lines). The number of observed arrangement types has not reached a plateau, and will likely increase as more host-specific *Salmonella* strains are characterized.

While each of the analyzed host-specific serovars has a most common arrangement type, a number of strains in each serovar have unique arrangement types. However, out of the 1,440 possible *rrn* arrangement types that can occur, only 48 have been identified so far in naturally-occurring strains of host-specific *Salmonella* serovars. How much diversity would be found if the arrangement types of more strains were determined? An arrangement type accumulation curve (Figure 3) and the estimation of the arrangement type richness computed using EstimateS ver. 8.2 [51] suggest that an additional 196 arrangement types (with a 95% confidence interval of 49–783 additional arrangement types) occur naturally in host-specific *Salmonella* serovars. If this estimate is correct, more than 80% of possible arrangement types do not occur naturally.

Selection appears to prevent certain arrangement types from becoming fixed within a population. If replichore balance is a major selective force in determining naturally-occurring arrangement types, most natural arrangement types would be predicted to have well-balanced replichores, and arrangement types with imbalanced replichores would be rare. While physical mapping data supports this idea [27], to fully understand how much of a selective force replichore balance truly is, one must know the putative replichore balance of arrangement types not observed in isolated strains.

### Estimated Replichore Balance of All Possible Arrangement Types

To determine the replichore balance of arrangement types not occurring in nature, as well as to quickly estimate the replichore balance of strains with established arrangement types, a replichore balance calculator was written in PERL. Physical mapping can detect strain-specific differences in replichore length due to various insertions and deletions [27], however the variability in replichore balance between strains with the same arrangement type has not been determined. The calculator described here allows a rapid estimation of replichore balance for all arrangement types, both natural and theoretical.

To compensate for the variation in the size of the chromosomal regions between the *rrn* operons, an average of region sizes from sixteen sequenced *Salmonella* strains representing both broad host range and host-specific serovars was used in the replichore balance calculations (Table 3). The origin of replication and *dif* were used as replichore endpoints. The origin of replication was placed 16 kilobasepairs (kbp) upstream of *rrnC*, between the *gidA* and *mioC* genes. The *dif* site was identified in each strain based on homology to the *Escherichia coli dif* site (accession number S62735; [52]). The distance between the 3′ end of *rrnG* and *dif* ranged from ∼550 kbp in Paratyphi C RKS4594 to 1,245 kbp in Typhi CT18, and averaged 1,117 kbp. The smaller distance observed in Paratyphi C RKS4594 is due to an inversion between the Gifsy phages, which causes this strain to be highly imbalanced [35]. Since this rearrangement skews the normal distance between the 3′ end of *rrnG* and *dif*, the data from this strain was not used. The mean distance between the 3′ end of *rrnG* and *dif* used in the replichore balance calculations was 1,155 kbp.

**Table 3:** Size of regions between *rrn* operons of sequenced *Salmonella* strains.

Region size in basepairs									
Serovar	Accession #	1	2	3	4	5	6	7	Total
Typhimurium LT2	AE006468	2504388	770324	535983	96088	155071	43424	752154	4857432
Choleraesuis	AE017220	2533708	707552	508771	95087	155208	42757	712617	4755700
Typhi CT18	AL513382	2430061	705901	508423	134412	149001	42215	839024	4809037
Typhi Ty2	AE014613	2397285	716333	515429	134577	148993	42055	837289	4791961
Paratyphi A	CP000026	2326185	758888	496761	99133	150592	42103	711567	4585229
Paratyphi A	FM200053	2321680	759028	496762	99139	150595	42104	712489	4581797
Paratyphi B	CP000886	2525347	715196	514103	96762	151034	43014	813431	4858887
Paratyphi C	CP000857	2515255	704163	527042	94942	155197	42761	793720	4833080
Gallinarum	AM933173	2453569	686501	516254	94715	159261	43323	705074	4658697
Arizonae	CP000880	2404145	709018	456966	93506	140141	45956	751068	4600800
Enteritidis	AM933172	2459619	699795	516880	94413	158527	43454	713160	4685848
Agona	CP001138	2416162	772925	529816	97977	154169	42905	784706	4798660
Dublin	CP001144	2590573	732200	515919	94350	159145	42915	707806	4842908
Heidelburg	CP001120	2512927	776445	523741	94412	143500	42144	795599	4888768
Newport	CP001113	2512812	749841	512410	94381	181958	42061	734178	4827641
Schwarzengrund	CP001127	2462704	715597	506261	98279	143900	42341	739993	4709075
**Average:**		**2460401**	**729982**	**511345**	**100761**	**153518**	**42846**	**756492**	**4755345**
Standard deviation:		74977	29790	18024	13293	9462	971	47527	104318

**Figure 4:**
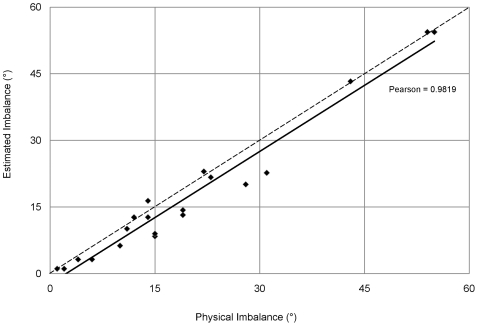
Estimated vs. physical replichore balance. While the calculator slightly underestimated replichore balance (dashed line  =  perfect fit), the estimates of replichore balance were statistically the same as the physically mapped arrangement types (*P*<0.01, paired Student's *t* test; Pearson's correlation  = 0.982).

To validate the calculator, balance estimates were generated using the average region sizes of the sequenced Typhi strains CT18 and Ty2 [33], [53] and compared to the balance calculated from the physical mapping of 29 Typhi strains with unique arrangement types [27] (Figure 4). Replichore imbalance in these arrangement types varied from 1–55°. While the calculator slightly underestimated the physical balance, mostly due to strain-specific increases in region size from insertions, the balance estimates from the calculator statistically agree with the physical balance of these arrangement types (*P*<0.01, paired Student's *t* test; Pearson's correlation  = 0.982).

**Figure 5:**
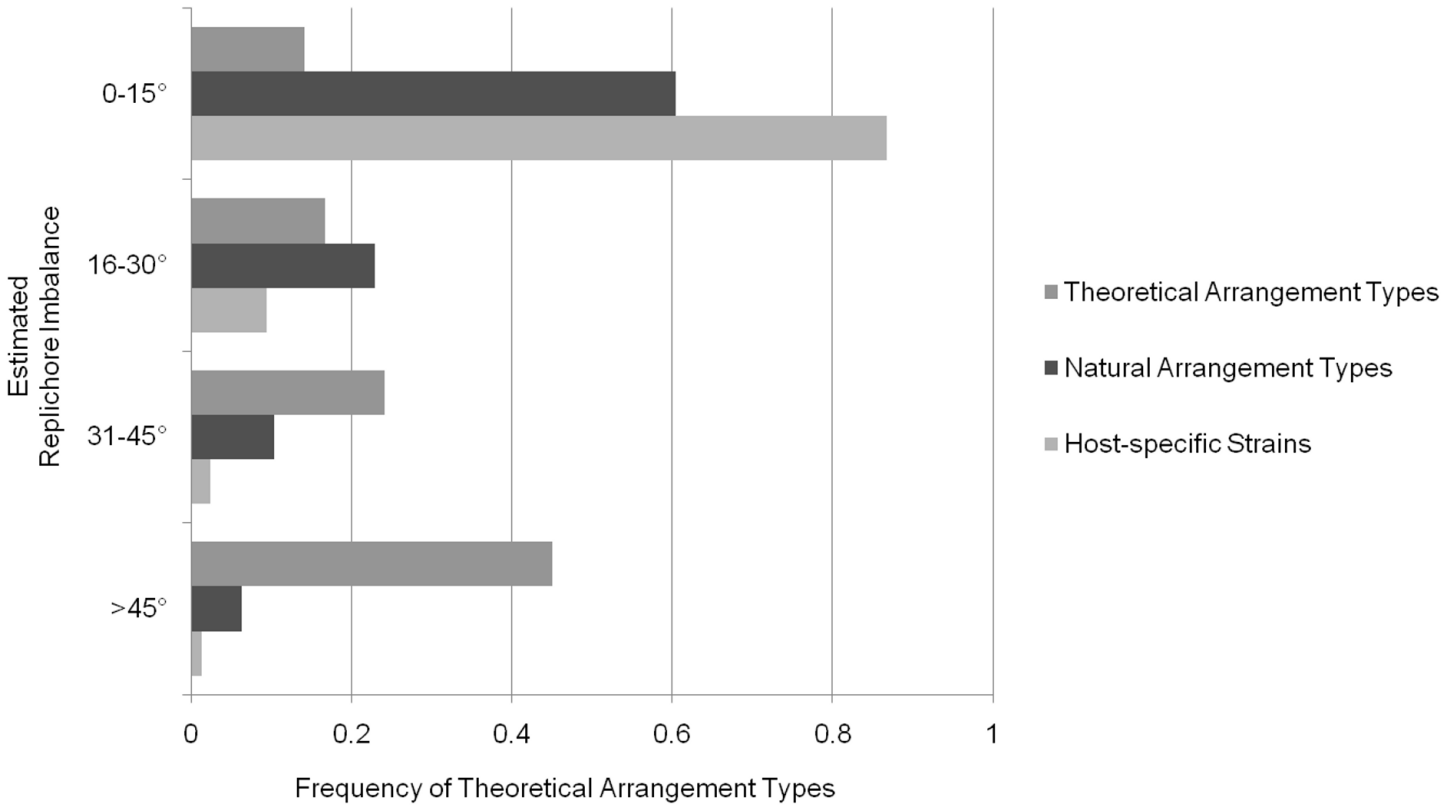
The estimated replichore imbalance of theoretical arrangement types, natural arrangement types, and host-specific strains. Estimated replichore imbalance was divided into four groups with every 15° increase of imbalance, and compared to the frequency of theoretical arrangement types. Most host-specific strains and natural arrangement types had well-balanced replichores (<15° imbalance), whereas most theoretical arrangement types had imbalanced replichores (>30° imbalance).

The replichore balance of the 48 naturally-occurring arrangement types was estimated (Figure 5; Table S1). Most naturally-occurring arrangement types have well-balanced replichores, with 29 observed arrangement types having≤15° imbalance. This group includes the conserved arrangement type with an estimated 0.2° imbalance. Another 11 naturally-occurring arrangement types have an imbalance of 16–30°, and 5 arrangement types have between 31–45° imbalance. Only 3 naturally-occurring arrangement types have an estimated replichore balance >45°. The 48 naturally-occurring arrangement types analyzed here were identified from 212 host-specific strains belonging to mostly the Typhi serovar [40], [44], [54], as well as the Gallinarum [36], [39], [41], [49], Paratyphi A [45], and Paratyphi C [42] serovars. Well-balanced replichores (≤15° imbalance) were estimated in 184 of these strains, and 20 strains had an imbalance between 16–30°. Five strains had between 31–45° imbalance, and only 3 strains had >45° imbalance. These results agree with previous results that naturally-occurring strains of bacteria often have well-balanced replichores [26], [27].

On the other hand, when the replichore balance of all 1,440 possible arrangement types was estimated, only 204 arrangement types (14% of total) were well-balanced (≤15° imbalance) (Figure 5; Table S2). Most possible arrangement types appeared to be very imbalanced. The number of arrangement types having an imbalance between 31–45° was 348 (24% of total), and 648 (45% of total) were >45° imbalanced (a third of these were >60° imbalanced). Another 240 arrangement types (17% of total) had an imbalance between 16–30°. These results show that rearrangements would most likely lead to a less balanced state, and that there are 175 balanced arrangement types that were not found among the naturally-occurring arrangement types. Over 90% of the non-natural, well-balanced arrangement types had at least one region translocated between Region 1 and 2 and/or Region 7 and 1. This is in contrast to only 3 naturally-occurring arrangement types with this configuration. These data suggest that other factors such as chromosomal location of a region can limit genome plasticity in addition to, if not more than, replichore balance.

## Discussion

In contrast to the conserved arrangement type observed in broad host range serovars of *S. enterica*, strains belonging to host-specific serovars almost always have chromosomal rearrangements from recombination between *rrn* operons. The Liu hypothesis proposes that rearrangements occur to reestablish replichore balance after horizontal gene transfer events [27], [38], [41]. To test this hypothesis, the naturally occurring arrangement types were compared to the theoretical possibility, and the replichore balance of these arrangement types was estimated.

As predicted by the physical mapping data [27], most arrangement types and almost all host-specific strains have well-balanced replichores. This observation has been suggested to be due to rearrangements reestablishing balance after insertions and deletions, as predicted by the Balanced Replichore hypothesis [27]. Wavelet analysis and bipartition modeling of numerous sequenced bacterial strains showed a strong tendency towards balanced replichores [28], [29], and it has also been suggested that imbalanced replication is detrimental to fitness and strains with this defect are selected against and lost from the population [22], [26], [30]. This argues that most strains are well-balanced because selective forces remove imbalanced strains versus imbalanced strains undergoing rearrangements to become balanced.

The most common rearrangement found in this analysis was an intrareplichore translocation of Regions 4, 5, and/or 6. However, this type of rearrangement has no effect on replichore balance. Furthermore, both the inversion of Region 1, the other common rearrangement, as well as the inversion from recombination between *rrnD* and *rrnE*, are symmetrical and have a negligible effect on balance. These observations that most rearrangements do not affect balance do not support the Balanced Replichore hypothesis.

Only 48 out of 1,440 arrangement types have been found to occur naturally. While an arrangement type accumulation curve (Figure 3) shows that other arrangement types will most likely be found in the future as more strains are analyzed, currently over 95% of theoretical arrangement types have not been observed. One explanation for this is that almost half of theoretical arrangement types are very imbalanced (>45°), and 70% have >30° imbalance. If most arrangement types are imbalanced, how probable is it that a rearrangement would increase balance, especially if the initial imbalance was caused by an insertion as proposed by the Balanced Replichore hypothesis? Insertions of pathogenicity islands or prophages into the *Salmonella* chromosome vary in size from 15–140 kbp, and would introduce up to 10° imbalance. However the chromosomal rearrangements occurring in host-specific *Salmonella* would most likely further increase this amount of imbalance rather than decrease it. This observation also does not support Liu's hypothesis.

Most DNA replication forks in *E. coli* and *Salmonella* are presumed to terminate in the replication fork trap between the *terC* and *terA* sites [55], [56], [57]. As the fork trap in *Salmonella* is almost 200 kbp in size, imbalance should be buffered up to 15°. Even slightly higher amounts of imbalance would be buffered by the *terD* and *terB* sites flanking the primary fork trap. Rearranging the chromosome in an attempt to correct this amount of imbalance would most likely introduce more imbalance as the amount of imbalance introduced from horizontal transfer of pathogenicity islands and prophages is buffered by the size of the fork trap. In contrast, rearrangements such as interreplichore translocations and asymmetrical inversions can easily introduce >15° imbalance.

In addition to replichore balance, gene location can influence genome plasticity. Positional effects on genome plasticity independent of replichore balance are evident in the well-balanced, non-natural arrangement types. Only 15 out of 175 such arrangement types have Region 1 flanked by Regions 2 and 7. However, 92% of naturally-occurring arrangement types have this configuration. This observation suggests a strong selection against arrangement types where Regions 4, 5, or 6 flank Region 1.

Changing the chromosomal address of a gene can affect its replication-associated copy number, or gene dosage. Rearrangements can change the dosage of genes in Regions 4, 5, and 6 by moving them farther from the origin of replication, for example into *rrnG* or *rrnH* flanking Region 1. Many genes in these regions encode proteins involved in the transcription and translation machineries, and gene dosage effects on expression have been suggested to limit their chromosomal location close to the origin of replication [18]. Within the observed naturally-occurring arrangement types, only five have Region 4, 5, or 6 flanking Region 1, and these arrangement types are each represented by only one strain.

Location may also prevent certain arrangement types from naturally occurring if macrodomain organization is perturbed. Recent studies in *E. coli* have described the structure of the chromosome in terms of four macrodomains and two non-structured regions that are spatially and temporally separated within the cell [22], [25], [58], [59]. Regions 4, 5, and 6, as well as part of Region 3 and most of Region 7 lie within the Ori domain. Analyzed inversions between the Ori and Left domains are interreplichore, asymmetrical, and introduce significant imbalance, which may mask the effect of mingling macrodomain-specific sequences. Intrareplichore inversions with endpoints in the Ori and Right macrodomains do not change balance but do often cause growth defects by interfering with nucleoid management and septum formation. If the *Salmonella* chromosome has a similar macrodomain structure, does that play a role in limiting the arrangement types observed? Since the *rrnH* operon is in the right non-structured region, rearrangements should be tolerated there. However as the *rrnG* operon is in the Left domain, translocation of Ori domain regions may be selected against. Only one strain analyzed in this study, a Paratyphi C strain, had such a translocation.

The number of recombination steps required to obtain certain arrangement types may also limit which arrangement types naturally occur. However this is unlikely as any of the 1,440 theoretical arrangement types can be obtained with a maximum of 3 recombination events, including many of the naturally-occurring arrangement types.

When naturally-occurring arrangement types were separated into their respective serovars, it was found that each serovar had its own most common arrangement type. In the most common Typhi arrangement type 1′235647, Region 4 translocated between Regions 6 and 7 and Region 1 was inverted relative to the conserved arrangement type. The next most common arrangement type had the same translocation of Region 4, but lacked the inversion and was more balanced. In Paratyphi C, the most common arrangement type 1263457 had an interreplichore translocation of Region 6, which slightly altered balance about 3°. The two biovars of the Gallinarum serovar differed in respect to their most common arrangement type. Although the Pullorum biovar had a most common arrangement type of 1735462, none of the analyzed Gallinarum biovar strains had that arrangement type. Furthermore, a most common arrangement type was not observed for the Gallinarum biovar, possibly due to the sample size. Interestingly two Gallinarum strains had the conserved arrangement type, which is very rare in host-specific *Salmonella* strains. Multilocus enzyme electrophoresis [60] and comparative genome analysis [36] have suggested that Gallinarum is a recent descendant of the Enteriditis serovar, which also has the conserved arrangement type. While the Pullorum and Gallinarum biovars are closely related [60], [61], the observed arrangement types in Gallinarum are more ancestral than the ones found in the Pullorum. Furthermore, the 1735462 arrangement type has undergone two inversions, one between *rrnG* and *rrnH* and one between *rrnD* and *rrnE*. The *rrnD*-*rrnE* inversion is interesting because the same inversion in *E. coli* is rapidly overgrown in culture by revertants, suggesting that it causes a fitness defect [30]. In Pullorum this inversion has not only persisted, but appears to be preferred. In spite of having a most common arrangement type, unique arrangement types were found in many strains, showing that diversity in arrangement types occurs within a serovar.

In conclusion, the results of this study do not support the hypothesis proposed by Liu that replichore imbalance drives the chromosomal rearrangements in host-specific *Salmonella* serovars. The effects on fitness due to changes in replichore balance from horizontal gene transfer is negligible because the replication fork trap where DNA replication terminates is large enough to buffer the imbalance introduced by known horizontal gene transfer events. Also most natural rearrangements in host-specific *Salmonella* do not significantly alter replichore balance while most theoretical arrangement types are very imbalanced. Therefore it seems unlikely that these types of rearrangements would improve balance over time.

An alternative possibility to that proposed by Liu is that lifestyle differences of the host-specific serovars are either inducing the rearrangements by increasing recombination frequency, or decreasing selective pressure to maintain gene order. One lifestyle difference is that host-specific serovars often establish a chronic carrier state within their hosts, usually residing within macrophages. Macrophages kill bacterial pathogens with bursts of reactive oxygen and nitrogen species produced by phagocyte NADPH oxidase and iNOS (inducible nitric oxide synthase) respectively. These bursts of reactive species have been shown to kill or inhibit intracellular *S. enterica* sv. Typhimurium *in vitro*, and are required for host resistance to infection [62], [63], [64]. However, the protein effectors encoded in *Salmonella* pathogenicity island-2 (SPI-2) enable intracellular *Salmonella* to resist these bursts of reactive species by preventing colocalization of the NADPH oxidase and iNOS with the *Salmonella*-containing vacuole (SCV) [65], [66], [67]. While the SPI-2 effectors provide protection from the reactive species bursts, over time in the carrier state the bursts are likely to occasionally hit the intracellular *Salmonella*. If DNA damage occurs and the DNA repair systems are induced, the rearrangements could be the result of increased recombination frequency. Although transcriptional profiling has shown the SOS response to be induced in Typhimurium cells isolated from infected J774-A.1 murine macrophage-like cells [68], there did not appear to be substantial upregulation of SOS genes in Typhi cells isolated from human THP-1 macrophages [69].

During establishment of the carrier state, small numbers of intracellular bacteria chronically colonize various host organs. Under these conditions the bacteria grow slowly, have less competition, and therefore may be under less selective pressure to maintain gene order. In addition, certain arrangement types may be selected for by the host. This would explain why each host-specific serovar has a most common arrangement type. Also, bottlenecks that occur during transmission to a new host are much narrower for host-specific serovars than for broad host range serovars, and may allow cells with rearrangements to become fixed within the population. These differences in lifestyle may also explain the rearrangements observed in strains belonging to host-specific *Salmonella* serovars and need to be further scrutinized.

## Materials and Methods

### Strains, growth conditions, and characterization

Strains used in this study are described in Table 4. Bacteria were cultured using Luria-Bertani (LB) medium at 30°C. Solid LB plates were prepared by adding agar to 1.5% (w/v). Motility was assessed as described in [70]. Serological identification was performed using *Salmonella* O antiserum for Group D_1_ Factors 1, 9, &12 (Difco, Detroit, MI, USA) preabsorbed to *S. enterica* Typhimurium LT2 to remove the α-Factor 1 and α-Factor 12 antibodies. Pullorum and Gallinarum biovars were distinguished by the ability to decarboxylate ornithine. Pullorum strains can rapidly decarboxylates ornithine whereas Gallinarum strains cannot [71].

**Table 4:** *Salmonella enterica* sv. Gallinarum strains analyzed in this study.

Strain	Alias	Biovar	Source^a–e^
TYT3313	RKS 4994	Gallinarum	SGSC
TYT3314	RKS 5079	Pullorum	SGSC
TYT3315	RKS 5021	Gallinarum	SGSC
TYT3316	SA 4404	Gallinarum	SGSC
TYT3325	SA 1684	Gallinarum	SGSC
TYT3326	SA 1685	Pullorum	SGSC
TYT3328	SA 1687	Pullorum	SGSC
TYT3329	SA 1688	Pullorum	SGSC
TYT3331	SA 1689	Pullorum	SGSC
TYT3335	TK619 ISM 1357	Gallinarum	ISU
TYT3339	X3796	Gallinarum	WU
TYT3340	X3544	Pullorum	WU
TYT3341	X3799	Pullorum	WU
TYT3342	X3539	Pullorum	WU
TYT3343	SEPRL #99	Pullorum	SEPRL
TYT3345	SEPRL #92	Pullorum	SEPRL
TYT3349	JEO 1911	Gallinarum	CGM
TYT3350	JEO 1909	Gallinarum	CGM
TYT3352	JEO 2555	Pullorum	CGM
TYT3353	JEO 2600 R9	Pullorum	CGM
TYT3354	JEO 2617 G200/81	Pullorum	CGM
TYT3355	JEO 2614	Pullorum	CGM

a **Salmonella Genetic Stock Center, University of Calgary, Calgary, Alberta, Canada;**

b **Iowa State University, Ames, IA;**

c **Washington University, Saint Louis, MO;**

d **US Department of Agriculture, Southeast Poultry Research Laboratory, Athens, GA;**

e **Centre de Génétique Moléculaire, Gif-sur-Yvette Cedex, France.**

### Isolation of chromosomal DNA

Chromosomal DNA was isolated using the Wizard® Genomic DNA purification kit as described by the manufacturer (Promega U. S., Madison, WI, USA).

### PCR conditions

Reactions were performed in HotStart 50 tubes (Molecular BioProducts, San Diego, CA, USA) and consisted of 200 µM dNTPs and 1 µM each primer (in the bottom layer), and 1× PCR buffer (20 mM Tris-HCL, pH = 8.4; 50 mM KCl; 0.8% Nonidet P-40), 1.25 mM MgCl_2_, 5% dimethylsulfoxide, Taq DNA polymerase isolated from *E. coli* harboring a plasmid with an inducible *taq* gene [72], and chromosomal DNA (in the top layer). Primer sequences and combinations for detecting specific *rrn* combinations were previously described [49]. Reactions were heated to 94°C for 3 min followed by 30 cycles of 94°C for 1 min, 60°C for 1 min, and 72°C for 5 min, followed by a final step at 72°C for 7 min. Presence of *rrn* PCR products were determined by running 10 µl of each reaction out on a 0.8% agarose/1× TBE gel, followed by detection using ethidium bromide staining.

### EstimateS

The freeware program EstimateS version 8.2 [51] was used to compute accumulation curves and estimate richness of naturally-occurring arrangement types. Sample order was randomized 100 times. Expected species richness values were computed using the moment-based estimator of species richness, τ(*h*), of naturally-occurring arrangement types, and a species accumulation curve was plotted with 95% confidence intervals as described in [73]. The Chao 2 [74] richness estimator in EstimateS was used to estimate the total number of naturally-occurring arrangement types using the classic formula as recommended.

### PERL calculator to estimate replichore balance

To estimate replichore balance, the size used for each chromosomal region between the *rrn* operons was the mean length of each region from sixteen sequenced *Salmonella* strains (Table 3). The origin of replication and the *dif* site were used as the replichore endpoints. The replichore balance calculator is available on the internet at http://edwards.sdsu.edu/cgi-bin/replichores.cgi.

## Supporting Information

Table S1 Estimated Replichore Length and Balance of Natural Arrangement Types.
(0.02 MB XLSX)

Table S2 Estimated Replichore Length and Balance of Theortetical Arrangement Types.
(0.09 MB XLSX)
